# The Positive Effects of One-Hour Intravenous Administration of Bortezomib on Peripheral Neuropathy in Multiple Myeloma Patients

**DOI:** 10.1155/2014/237698

**Published:** 2014-06-04

**Authors:** Joo Young Jung, Ho Young Kim, Boram Han, Dae Ro Choi, Dae Young Zang, Hyo Jung Kim

**Affiliations:** ^1^Department of Internal Medicine, Dongtan Sacred Heart Hospital, Hwaseong-si 445-907, Republic of Korea; ^2^Division of Hematology/Oncology, Department of Internal Medicine, Hallym University Sacred Heart Hospital, Hallym University College of Medicine, 22 Gwanpyeong-ro, 170 Beon-gil, Dongan-gu, Anyang-si 431-796, Republic of Korea; ^3^Department of Internal Medicine, Chuncheon Sacred Heart Hospital, Chuncheon-si 200-704, Republic of Korea

## Abstract

Bortezomib-induced peripheral neuropathy (BiPN) in multiple myeloma (MM) patients is a common and serious side effect. Currently, it has been reported that subcutaneous (SC) administration of bortezomib decreases the incidence of BiPN as compared to standard intravenous (IV) bolus injection without any differences in efficacy. However, there are reports of severe injection site reaction following SC administration of bortezomib. The aim of this study was to evaluate the response rate and incidence of BiPN following one-hour IV infusion of bortezomib. The data was retrospectively collected from MM patients who had been treated with IV administration of bortezomib for one hour. Twenty-three patients were evaluated (median age 72 years, 13 males). The median number of treatment cycles was 5 (range 2–10). The cumulative bortezomib dose was 26.0 mg/m^2^ (14.3–66.3) and percent of actual per expected cumulative dose was 90% (50–100). The overall response (complete response plus partial response) rate was 65%. The incidence of BiPN was 57% (*n* = 13) and incidence of severe neuropathy was 4% (*n* = 1). One-hour IV infusion of bortezomib was an effective regimen for MM with reduced incidence of severe BiPN. This route of administration of bortezomib could be an alternative mode of delivery for patients with severe injection site reactions following SC administration.

## 1. Introduction

With respect to patient outcomes in multiple myeloma (MM), there have been significant improvements after the discovery of novel induction agents, including bortezomib [1, 2]. However, intravenous (IV) bolus injection of bortezomib, which is the standard mode of administration has limitations because of bortezomib-induced peripheral neuropathy (BiPN) [3, 4]. BiPN is one of the most common, often reversible, but significant side effects of bortezomib therapy, which leads to dose modification [3–6]. The typical form of neuropathy is largely sensory rather than motor and is in the feet rather than in the hands. Although the pathogenesis underlying BiPN is not precisely understood, most investigators agree that bortezomib induces sensory nerve injury in a dose-dependent manner. Furthermore, the most significant risk factor of the onset or aggravation of BiPN is preexisting peripheral neuropathy.

Until now, the management of BiPN has consisted of dose reduction or discontinuation of IV bolus bortezomib. Recent reports have demonstrated that the incidence and severity of BiPN in patients receiving subcutaneous (SC) bortezomib are significantly lower than those in patients treated with an IV bolus of the drug, with noninferior efficacy in MM [7, 8]. This decreased incidence and severity of BiPN in the SC bortezomib group could be potentially due to the lower maximum plasma drug concentration (*C*_max⁡_) and longer time to reach *C*_max⁡_ (*T*_max⁡_) with the same area under the concentration (AUC) when compared to IV bolus bortezomib.

SC bortezomib also has a different weak point, the injection site reaction (ISR), because bortezomib itself is one of the irritant drugs and induces an inflammatory reaction. In most cases, the SC bortezomib-related ISR is well tolerated, but cases of severe skin reactions including necrosis have been reported [8, 9]. Therefore, a new route of administration is necessary, particularly in patients with severe ISR following SC administration of bortezomib. SC administration of bortezomib could not be applied to Korean MM patients, because this route has not been previously allowed by the Korean health insurance system until recently. Meanwhile, our group had attempted to increase the infusion time of bortezomib to one hour, in order to maintain AUC, without abrupt change of concentration of bortezomib, while decreasing *C*_max⁡_ and *T*_max⁡_ of the drug in MM patients.

In this study, we retrospectively evaluated the response rate of the drug and incidence of BiPN following one-hour IV infusion of bortezomib.

## 2. Patients and Methods

This study was designed to retrospectively review the medical records of patients with MM, who were treated with bortezomib-based regimen between January 2009 and March 2014 at Hallym University Sacred Heart Hospital. The route of bortezomib administration was IV infusion for one hour. Bortezomib was reconstituted with 100 mL of normal saline. The data of patients with any regimen containing bortezomib, any line of treatment, and previous treatment with bortezomib were included. Patients excluded from the study were as follows: (1) patients with dementia or other neurologic deficits which prevent expression of subjective neurologic symptoms, (2) patients who received less than 2 cycles of chemotherapy and could not measure the treatment response, and to these patients BiPN should not be the reason of treatment cessation, and (3) patients diagnosed with peripheral neuropathy grade 3 or more according to the National Cancer Institute Common Terminology Criteria for Adverse Events (CTCAE) ver4.0 [10], before starting the bortezomib-based chemotherapeutic regimen.

The primary end points of this study were the incidence and severity of BiPN according to the CTCAE ver4.0. Assessment of CTCAE grades of BiPN was dependent on the subjective symptoms of patients, physical examination by attending physicians, and medication history. The secondary end points were the cumulative dose and duration of bortezomib, the best response rate, and survival. The expected cumulative dose of bortezomib was calculated based on the dose of the drug at VISTA trial [11] when patients were treated with velcade, melphalan, and prednisolone (VMP) regimen. The dosage was calculated based on 1.3 mg/m^2^ at days 1, 4, 8, and 11 per one cycle for other bortezomib-containing regimens. The dose modification of bortezomib was performed according to the package insert of the drug [12] depending on the adverse events of patients. Disease response was assessed with criteria from the International Myeloma Working Group (IMWG) [13, 14]. However, not all patients with negative serum and urine protein electrophoresis and immunofixation results underwent bone marrow biopsy to confirm complete response (CR) in routine clinical practice. We incorporated near complete response (nCR) [14] as a response measurement. The reimbursement policy of Korean National Health Insurance Service supports bortezomib treatment only if the response is partial response (PR) or better at the end of four cycles; patients with minimal response (MR) or stable disease (SD) had to discontinue the bortezomib-based treatment. Therefore, we incorporated MR [15] to subdivide the response measurement, in order to know the status of disease at the time of drug cessation. We defined progression free survival (PFS) as the time from initiation of treatment to disease progression, relapse, or death from any cause or initiation of the subsequent chemotherapy, whichever came first. Overall survival was defined as the time from initiation of treatment to death from any cause.

Survival was analyzed using the Kaplan-Meier methods, and statistical analyses were performed with IBM SPSS (version 21). The Institute Review Board of Hallym University Sacred Heart Hospital approved the protocol of this retrospective study.

## 3. Results

Twenty-three Korean patients with MM were enrolled in this study. All patients had measureable disease. Patient characteristics and demographics are summarized in Table 1. Median age was 72 years. Eleven were newly diagnosed MM (NDMM) patients and treated with VMP regimen. Twelve were relapsed or refractory MM (RRMM) patients to previous lines of chemotherapy, and among them 3 patients had been exposed to bortezomib. Various bortezomib-containing regimens were administered to these patients (Table 2). The total cycles of chemotherapy regimens were 78 cycles, and the median number of cycles per patient was 5 cycles with the median treatment duration of 19 weeks. The median cumulative dose of bortezomib was 26 mg/m^2^ (range 14–66 mg/m^2^) and the percent intensity of actual per expected cumulative dose of each regimen was 90% (range 50–100%). Seven patients (31%) discontinued therapy because of disease progression. Four patients (17%) with MR or SD, at the end of the 4th cycle of chemotherapy, had to finish the current treatment.

**Figure 1:**
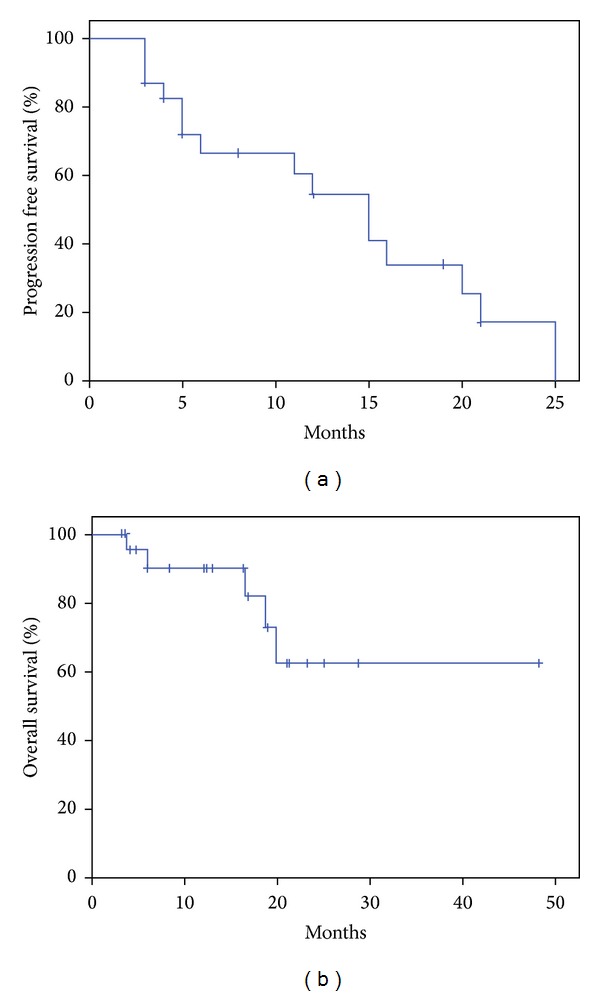
Progression free survival (a) and overall survival (b).

The best overall response rate (ORR, from PR to CR) during treatment was 65% (Table 3), including 1 (4%), 2 (9%), and 6 (26%) patients with CR, nCR, or VGPR, respectively. And the best ORR of NDMM and RRMM group was 55% and 75%, respectively (*P* = 0.4). After a median follow-up of 16.3 months (interquartile range 6.0–21.0), 5 patients (21.7%) had died and median overall survival was not reached (Figure 1). Causes of death were infection (*n* = 3) and progression of MM (*n* = 2). Median PFS was 15.0 months (95% CI 10.2–19.8), and 1-year PFS rate was 54.4% (95% CI 31.9–76.9).

Table 4 shows the safety profiles of intravenous bortezomib infusion treatment. Five patients (22%) discontinued therapy because of adverse events, but peripheral neuropathy was not a cause for cessation of bortezomib therapy for any patient. Anorexia, peripheral sensory neuropathy, upper respiratory infection, and nausea were the most common adverse effects and few patients suffered grade 3 toxicities. However, hematology laboratory data showed 39%, 35%, 43%, and 26% of grade 3 or more results on hemoglobin, WBC, absolute neutrophil count, and platelet, respectively.

Four out of 23 patients had peripheral neuropathy with grade 1 (*n* = 2) or 2 (*n* = 2) before initiation of bortezomib treatment. The grade 2 neuropathy of a patient was exacerbated to grade 3 during bortezomib therapy, but it turned to grade 2 after cessation of bortezomib. There was no change on the severity of neuropathy of the other 3 patients during chemotherapy. Rates of peripheral neuropathy events of grades 1, 2, and 3 severity were 43.5% (*n* = 10), 8.7% (*n* = 2), and 4.3% (*n* = 1), respectively. Among 13 patients with peripheral neuropathy, 7 were NDMM patients with grade 1 (*n* = 5) or 2 (*n* = 2) severity. Six patients were RRMM patients with grade 1 (*n* = 5) or 3 (*n* = 1) severity. The estimated time to onset of peripheral neuropathy was 4.6 months (95% CI 1.1–8.0) from initiation of bortezomib treatment. There was no significant difference of this time between NDMM and RRMM patients (4.6 months, 95% CI 3.0–6.1 and 6.5 months, 95% CI 0–14.6, resp.). Cumulative dose of bortezomib at the first onset of peripheral neuropathy was 16.5 mg/m^2^ (95% CI 9.2–23.6). There was no significant difference of cumulative doses at the end of bortezomib treatment between patients with and without peripheral neuropathy (*P* = 0.13).

## 4. Discussion

Bortezomib, a proteasome inhibitor, has improved the response and survival of MM patients [1, 2, 11]. This study showed that one-hour IV infusion of bortezomib was an effective regimen for MM with reduced incidence of severe BiPN. The patient population of this study was heterogeneous. Both NDMM and RRMM patients were evaluated with various bortezomib-containing chemotherapeutic regimens and schedules. Drug cessation was defined not only by response or adverse reactions but also by insurance guidelines. Under these circumstances, we decided to use the best overall response rate for measurement of effectiveness. Compared with 71% in the VISTA trial for NDMM patients [11] and 38% in the APEX trial for relapsed MM patients [16], the best overall response rate of our trial was 65%. Taken together, with 17% of patients (*n* = 4) who had to finish the drug to follow insurance guidelines, even with the response of MR or SD at the end of 4 cycles, one-hour IV infusion did not seem to affect the efficacy of bortezomib.

Several studies have reported that severe BiPN affects the quality of life of patients receiving bortezomib and could be a hurdle to continuation of the drug, even in patients with a good response [3–6]. The incidence of BiPN has been reported in up to 70% of patients, including grade ≥3 BiPN in up to 16%, increased with cumulative dose of standard twice-weekly 1.3 mg/m^2^ dose schedule through IV bolus administration [5]. In the VISTA trial [11], the incidence of grades 1, 2, 3, and 4 sensory BiPN was 14%, 17%, 13%, and <1%, respectively, in NDMM patients on the VMP regimen. Moreau et al. [17] reported 70% of any grade BiPN in the bortezomib and dexamethasone treatment group, which was used for induction before autologous stem cell transplantation in NDMM patients. Furthermore, the rates of grade 2 or worse and grade 3 BiPN were 34% and 11%, respectively. Bringhen et al. [18] reported that the incidence of grade 3/4 BiPN was significantly decreased, yet not completely resolved, in the once-weekly scheduled bortezomib group compared with the twice-weekly group. Currently, the MMY-3021 trial [8] shows that SC administration is not inferior in efficacy to IV bolus administration with 1.3 mg/m^2^ twice-weekly bortezomib in relapsed MM patients and significantly reduces the incidence of BiPN of any grade (38% versus 53%), ≥grade 2 (24% versus 41%), and ≥grade 3 (6% versus 16%). In our study, the incidence of BiPN was not reduced (57%); however, most cases of BiPN were grade 1 (44%) and did not require special management. And the rates of grade 2 or worse and grade 3 BiPN were low, only 13% and 4%, respectively, and BiPN did not necessitate cessation of the drug. In addition, cumulative dose of bortezomib was comparable to other prospective studies with the standard dose schedule. Therefore, in conjunction with dose or schedule modification and SC administration, one-hour infusion of bortezomib could be an additional option to reduce severe BiPN.

The MMY-3021 trial [8] also reported pharmacokinetic and pharmacodynamic evaluation of bortezomib administered through SC and IV bolus routes, and *C*_max⁡_ was ten times lower in the SC group than in the IV bolus group, with longer *T*_max⁡_ and similar AUC. Theoretically, one-hour IV infusion would decrease *C*_max⁡_ and prolong *T*_max⁡_. Until now, there has been no conclusive evidence of the correlation between these pharmacokinetic results and the low incidence of BiPN. However, this could be a potential explanation for the decrease in BiPN with SC administration and the one-hour IV infusion method, and further evaluation is warranted.

The SC mode of delivery of bortezomib is now indicated for use in the clinic in the EU, USA, and recently in Korea. Barbee et al. [19] surveyed MM patients in the clinic regarding their preferences on the route of administration between SC and IV bolus. Interestingly, not all patients preferred SC. A quarter of the patients preferred IV bolus, and one patient switched from SC to IV. Their reason for favoring IV was mainly injection site reactions (ISRs) of SC administration, such as bruising and pain. The reported incidences of bortezomib-related ISRs of all grades were variable up to 39% [9]. Most common reaction was erythema and bruising, and ISRs were usually well tolerated, resolving completely. However, 1% of patients suffered from severe ISRs [8]. There was a case of necrotizing skin eruption on the whole abdomen after SC administration of bortezomib [9]. Cautious monitoring is crucial at each SC administration.

Limitations of this study include a small sample size, single-center data, and the retrospective nature. Matsuoka et al. [20] have presented a small retrospective study about the incidence and severity of BiPN in patients who had been treated with three-hour intravenous infusion of bortezomib. Incidences of grades 1 and 2 BiPN were 30% and 15% of patients, respectively, and there were no patients with grade 3/4 BiPN. These results are very similar to our data and appear to support our conclusion. However, to confirm these observations, prospective study with pharmacokinetic and pharmacodynamic evaluation is needed.

## 5. Conclusion

The current study showed that one-hour IV infusion of bortezomib-containing regimen was an effective treatment for multiple myeloma. This administration route did not reduce the incidence of bortezomib-induced peripheral neuropathy but seemed to decrease the severity of peripheral neuropathy. This route of administration of bortezomib could be a promising alternative to subcutaneous administration, particularly in patients with severe injection site reactions.

## Figures and Tables

**Table 1:** Patients baseline characteristics.

	*N* = 23
Age (years)	72 (43–80)
Sex (male/female)	13 (56)/10 (44)
Diabetes mellitus	6 (26)
Time since diagnosis of MM (months)	1.9 (0–64.4)
Previous lines of treatment	
0	11 (48)
One line	11 (48)
VAD	1
BTD followed by MPT	2
High dose dexamethasone	3
Thalidomide + dexamethasone	2
Lenalidomide + dexamethasone	2
Cyclophosphamide + prednisolone	1
Two lines	1 (4)
Melphalan + prednisolone → bortezomib	
MM type	
IgG	14 (61)
IgA	5 (22)
Light chain	4 (17)
ISS stage	
I	8 (35)
II	6 (26)
III	9 (39)
Beta-2-microglobulin (mg/L)	4.6 (2–17.5)
Albumin (g/dL)	3.5 (2.2–4.6)
GFR (mL/min/1.74 m^2^)	74.3 (15.1–151.7)
Hemoglobin (g/L)	98 (64–130)
White blood cell (×10^9^/L)	4.9 (1.3–8.5)
Platelet (×10^9^/L)	171 (53–408)

Data are median (range) or number (%). GFR was calculated with MDRD equation. MM: multiple myeloma, VAD: vincristine + doxorubicine + dexamethasone, BTD: bortezomib + thalidomide + dexamethasone, MPT: melphalan + prednisolone + thalidomide, ISS: international staging system, and GFR: glomerular filtration rate.

**Table 2:** Treatment exposure.

	*N* = 23
Regimen	
VMP	13 (57)
VD	8 (35)
PAD	1 (4)
Bortezomib	1 (4)
Number of treatment cycles	5 (2–10)
Time on treatment (weeks)	19 (7–54)
Bortezomib cumulative dose (mg/m^2^)	26.0 (14.3–66.3)
Bortezomib dose intensity (mg/m^2^/cycle)	6.5 (2.6–10.4)
Bortezomib real/expected cumulative dose (%)	90 (50–100)
Cause of treatment cessation	
Progressive disease	7 (31)
Adverse events except peripheral neuropathy	5 (22)
Insurance reimbursement issue	4 (17)
On treatment	3 (13)
Patients refusal	3 (13)
Preplanned cycles	1 (4)

Data are median (range) or number (%). VMP: bortezomib, melphalan, and dexamethasone, VD: bortezomib and dexamethasone, and PAD: bortezomib, doxorubicin, and dexamethasone.

**Table 3:** The best response rate.

	*N* = 23	Rate (%)	Cumulative rate (%)
CR	1	4	4
nCR	2	9	13
VGPR	6	26	39
PR	6	26	65
Minimal response	3	13	78
Stable disease	3	13	91
Progressive disease	2	9	100

CR: complete response, nCR: near complete response, VGPR: very good partial response, and PR: partial response.

**Table 4:** Adverse events.

	All grades		Grade ≥ 3	
Anorexia	15	(65%)	0	
Peripheral sensory neuropathy	13	(57%)	1	(4%)
Upper respiratory infection	11	(48%)	1	(4%)
Nausea	11	(48%)	0	
Fatigue	8	(35%)	0	
Gastrointestinal pain	7	(31%)	0	
Diarrhea	7	(31%)	1	(4%)
Skin rash	5	(22%)	0	
Dizziness	5	(22%)	0	
Constipation	5	(22%)	0	
Headache	4	(17%)	0	
Vomiting	3	(13%)	0	
Cramping	3	(13%)	0	
Myalgia	3	(13%)	0	
Insomnia	3	(13%)	0	
Neuralgia caused by herpes zoster	3	(13%)	0	
Fever	3	(13%)	0	
Dyspnea	3	(13%)	0	
Noncardiac chest pain	3	(13%)	0	
Hematology laboratory data				
Hemoglobin	11	(48%)	9	(39%)
White blood cell count	19	(83%)	8	(35%)
Absolute neutrophil count	17	(74%)	10	(43%)
Platelets	20	(87%)	6	(26%)
